# Factors associated with post-COVID-19 confinement vape use in young adults: Josue Eli Villegas Dominguez

**DOI:** 10.1093/eurpub/ckac131.099

**Published:** 2022-10-25

**Authors:** JE Villegas Dominguez, C Reyes Hernandez, AR Rodríguez Barranco, M Pérez Santamaria, H Juárez de la Huerta, A Martínez Torralva, EA Gonzalez David, BL Muñoz Bautista, FG Marquez Celedonio

**Affiliations:** 1 Facultad de Medicina, Campus Veracruz, Universidad Veracruzana, Veracruz, Mexico; 2 Facultad de Medicina, Universidad del Valle de Mexico, Veracruz, Mexico

## Abstract

**Introduction:**

The use of Vape has increased during the pandemic due to the changes generated by it. Currently we have finished the conditions of confinement, so it is important to identify

Objective: To determine the factors associated with post-COVID-19 confinement vape consumption in young adults

**Methods:**

A cross-sectional, prospective study was carried out between January and April 2022, including men and women residents of Veracruz aged between 18 and 35 years, excluding participants with addiction treatment or with a diagnosis of a lung disease. A survey was applied through Google Forms to identify the factors associated with Vape consumption after confinement by COVID-19, including depression, anxiety and stress evaluated with the DASS-21 instrument (Cronbach’s alpha between 0.79 and 0.87). For data analysis, SPSS v22 software was used, X2 test with Odds Ratio (OR) and 95% confidence interval (CI95%) and MannWhitney U test, assigning statistical significance with p < 0.05.

**Results:**

514 participants were included, with a prevalence of vape use of 28.5%. Physical activity, cigarette consumption by a family member, levels of anxiety, depression and stress showed a value of p > 0.05 for Vape consumption, while other factors (OR/95%CI) such as being female (0.6/0.4-0.9), identifying vape advertising on Facebook (0.35/0.19-0.65), having a family member who vapes (2.4/1.5-3.6), consuming cigarettes (4.2/2.7-6.4) and identifying vape advertising on Instagram (1.5/ 1.0-2.3) had values of p < 0.05

**Conclusions:**

Post-pandemic vape use is not affected by anxiety, stress or depression, identifying other factors that favor its use such as the environment in which it develops, such as being someone with a history of tobacco use, in addition to the family smoker and advertising on Instagram, which is a social network that often works as an aspirational image for users, a situation contrary to what is shown on Facebook.

**Key messages:**

• Vape consumption continues to be a post-pandemic public health problem, so it is necessary to reinforce educational measures on the subject and the post-Covid complications of its use.

• It is necessary to regulate vape advertising on social networks, as has been done with tobacco in different countries, since it can become a risk factor by showing aspirational images for users.

